# Prediction of biodiversity hotspots in the Anthropocene: The case of veteran oaks

**DOI:** 10.1002/ece3.3305

**Published:** 2017-08-31

**Authors:** Olav Skarpaas, Stefan Blumentrath, Marianne Evju, Anne Sverdrup‐Thygeson

**Affiliations:** ^1^ Norwegian Institute for Nature Research Oslo Norway; ^2^ Natural History Museum University of Oslo Oslo Norway; ^3^ Faculty of Environmental Sciences and Natural Resource Management Norwegian University of Life Sciences Ås Norway

**Keywords:** forest, hollow oaks, land use change, landscape structure, large trees, presence‐absence, *Quercus petraea*, *Quercus robur*, species distribution modeling

## Abstract

Over the past centuries, humans have transformed large parts of the biosphere, and there is a growing need to understand and predict the distribution of biodiversity hotspots influenced by the presence of humans. Our basic hypothesis is that human influence in the Anthropocene is ubiquitous, and we predict that biodiversity hot spot modeling can be improved by addressing three challenges raised by the increasing ecological influence of humans: (i) anthropogenically modified responses to individual ecological factors, (ii) fundamentally different processes and predictors in landscape types shaped by different land use histories and (iii) a multitude and complexity of natural and anthropogenic processes that may require many predictors and even multiple models in different landscape types. We modeled the occurrence of veteran oaks in Norway, and found, in accordance with our basic hypothesis and predictions, that humans influence the distribution of veteran oaks throughout its range, but in different ways in forests and open landscapes. In forests, geographical and topographic variables related to the oak niche are still important, but the occurrence of veteran oaks is shifted toward steeper slopes, where logging is difficult. In open landscapes, land cover variables are more important, and veteran oaks are more common toward the north than expected from the fundamental oak niche. In both landscape types, multiple predictor variables representing ecological and human‐influenced processes were needed to build a good model, and several models performed almost equally well. Models accounting for the different anthropogenic influences on landscape structure and processes consistently performed better than models based exclusively on natural biogeographical and ecological predictors. Thus, our results for veteran oaks clearly illustrate the challenges to distribution modeling raised by the ubiquitous influence of humans, even in a moderately populated region, but also show that predictions can be improved by explicitly addressing these anthropogenic complexities.

## 1 INTRODUCTION

Global change implies an urgent need to better understand and assess the effects of human land management on biodiversity‐rich ecosystems and habitats (Erb et al., 2017; Souza, Teixeira, & Ostermann, 2015; Titeux et al., 2016). Concentrations of biodiversity can be found in many parts of the World (Gaston & David, 1994; Medail & Quezel, 1997; Myers, Mittermeier, Mittermeier, Da Fonseca, & Kent, 2000; Sverdrup‐Thygeson, Brandrud, & Ødegaard, 2007). Areas with a large number of species, especially rare, threatened or endemic species often occur in remote and relatively pristine natural areas, like tropical rain forest interior (Mittermeier, Myers, Thomsen, Da Fonseca, & Olivieri, 1998; Myers, 1988) and boreal old‐growth forests (e.g., Gjerde, Sætersdal, Rolstad, Blom, & Storaunet, 2004; Sverdrup‐Thygeson, Søgaard, Rusch, & Barton, 2014; Timonen, Gustafsson, Kotiaho, & Mönkkönen, 2011). However, areas with high biodiversity are not only confined to remote wilderness. In fact, there is often a high coincidence between people and biodiversity (Araújo, 2003). With the increasing presence and activities of humans, an increasing number of species‐rich habitats are found in ecosystems and biomes strongly influenced and transformed by us (“anthromes”; Ellis, Klein Goldewijk, Siebert, Lightman, & Ramankutty, 2010; Ellis & Ramankutty, 2008; Hobbs, Higgs, & Harris, 2009). From a conservation perspective, the most important biodiversity concentrations to keep an eye on are those under pressure, which has led some to include human‐induced threats in the definition of biodiversity hotspots (Myers et al., 2000). However, all human actions do not imply biodiversity loss. In Europe, some of the most species‐rich habitats outside the Mediterranean basin are seminatural grasslands, partly created and tended by humans today (Cremene et al., 2005). Similarly, veteran trees in Europe are often legacies from a preindustrial, extensively managed agricultural landscape, and owe some of their qualities to previous human management, like coppicing. Due to their rich microhabitat structures (thick bark, crevices, dead branches, hollows etc.), these veteran trees are not only important landscape elements, but often constitute local hotspots for biodiversity in themselves (Sverdrup‐Thygeson, 2009).

Biodiversity hotspots can be found at many spatial scales from global to local (Gaston & David, 1994; Medail & Quezel, 1997; Myers et al., 2000; Sverdrup‐Thygeson et al., 2007). While several global hotspots have been identified and roughly delineated geographically (e.g., Myers et al., 2000), locating local hotspots is more challenging. Yet finding these hotspots is critical for planning and management at the local level, where most decisions are made and land management is in action every day. Given that complete mapping is way beyond the resources allocated to biodiversity mapping and monitoring in most countries, some form of spatial distribution modeling (e.g., Elith & Leathwick, 2009) is needed. This is especially true in habitat types with a large suite of associated specialized species, where focusing on occurrence and critical properties of the habitat can be a cost‐efficient way of locating and protecting several species in one operation (Gjerde, Sætersdal, & Blom, 2007; Lehmann, Overton, & Austin, 2002; Skarpaas, Diserud, Sverdrup‐Thygeson, & Ødegaard, 2011). However, the mix of ecological and anthropogenic factors affecting local biodiversity hotspots poses several challenges to ecological and geographical prediction. There are many potentially important predictor variables, distributed across complex landscapes with gradients and thresholds in both ecological and anthropogenic influences over time (Erb et al., 2017). Now, at the beginning of the Anthropocene, there is a rapidly growing need to address these complexities.

**Figure 1:**
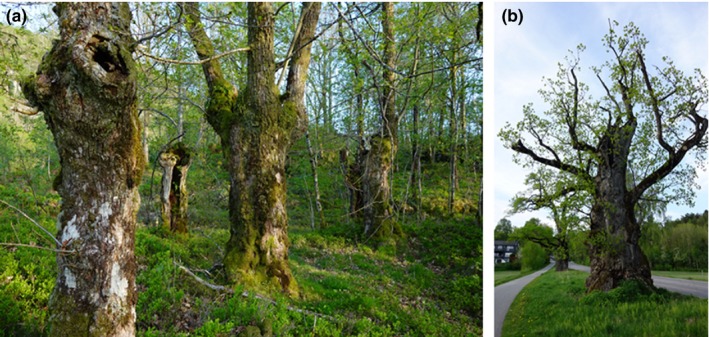
Veteran oaks in (a) forest (Mandal, Norway) and (b) open landscapes (Porsgrunn, Norway). Photos: Anne Sverdrup‐Thygeson

In this study, we focus on large and hollow oaks (*Quercus* spp.; Figure 1)—an important biodiversity hot spot habitat in northern Europe—to address the challenge of interacting ecological and anthropogenic processes in generating spatial patterns in biodiversity hotspots. Our goal is to develop robust process‐based predictions of hot spot oak occurrence, for use in conservation management and research. We concentrate on oaks in Norway, where large and hollow oaks (hereafter “veteran oaks”) were recently listed as a priority habitat under the Nature Diversity Act, and where comprehensive data sets are being collected as parts of national monitoring efforts and biodiversity studies (Sverdrup‐Thygeson, Evju, & Skarpaas, 2013).

Oaks are long‐lasting habitats, with some trees thought to be close to 1,000 years old (Drobyshev & Niklasson, 2010). As the years go by, the architectural diversity increases, and the oaks develop patches of decay, broken branches or flaking, deeply creviced bark—and after about 200 years of age, sometimes earlier, internal cavities start to develop (Ranius, Niklasson, & Berg, 2009). In these microhabitats, exceptionally species‐rich communities associated with wood decay and wood mold flourish. For these reasons, veteran oaks are a priority conservation habitat, and for both conservation research and management of these large old trees it is important to know where they are likely to be found and how they are influenced by ecological and anthropogenic processes (Lindenmayer & Laurance, 2016; Lindenmayer et al., 2014).

A critical question for the prediction of veteran oaks is how the distribution of these large and hollow oaks—the biodiversity hot spot oaks—differs from the distribution of oak in general. We know much about the distribution and ecology of oaks from previous studies (e.g., Annighöfer, Beckschäfer, Vor, & Ammer, 2015; Dahl, 1998; Jones, 1959; Stokland & Halvorsen, 2011). To what extent do veteran oaks follow the oak niche? Oaks grow old, large, and hollow when they have sufficient time to grow, age and decay without major disturbances (Ranius et al., 2009). Some 1,000 years ago, in a slightly warmer climate and before the impact of humans, oak forests covered large areas in southern Norway. In the 16th and 17th century, there was a high demand for oak timber for buildings and ships, and large amounts of Norwegian oak were exported to Europe (Moore, 2010; Vevstad, 1998). Today, only remnants of the previous oak forests are left. It is unclear, however, to what extent large and hollow oaks in forests are concentrated in remote, inaccessible and/or low‐productive areas. Veteran oaks are also found in open landscapes, and while certain aspects may be important in both open landscapes and in forests, such as the basic ecological requirements of oaks, other aspects, like human activities, may differ between landscape types. Moreover, the complexity of effects may be even higher in the open landscape, making predictions more difficult. A recent synthesis of ecological and historical knowledge of oak forests in North America suggest that humans may have played an important role in shaping the distribution and population structure of oaks, in interaction with several ecological factors, leading to the proposition of a “multiple ecosystem drivers hypothesis” of oak forest dynamics (McEwan, Dyer, & Pederson, 2011).

Given the complexity of interacting ecological and anthropogenic factors, how, and to what extent, can we predict the distribution of biodiversity hotspots in the Anthropocene? Our basic hypothesis is that even in moderately populated parts of the World, human influence is ubiquitous, and we make three predictions for Anthropocene biodiversity hot spot modeling that we test for the case of veteran oaks in Norway. We expect (i) human‐modified responses to ecological factors, (ii) fundamentally different processes and predictors in landscape types shaped by different land use histories, and (iii) a multitude and complexity of natural and anthropogenic processes that require many predictors and even multiple models. Specifically, for veteran oaks, we expect shifts in the realized niche toward inaccessible and low‐productive areas, and different responses to geographical and land cover variables in forests and open landscapes because of differences in past and present human land use. Moreover, because of the multiple ecological and anthropogenic drivers, we expect that the distribution of veteran oaks is better predicted with multiple predictors and models, combined in coherence with the structure of the landscape.

## 2 METHODS

### 2.1 Study system

Oak forests in Northern Europe are dominated by two oak species, sessile oak *Quercus petrea* and pedunculate oak *Quercus robur*. The two species frequently hybridize and may be difficult to separate in the field. Ecologically they are fairly similar, and we treat them together as oaks (*Quercus* spp.) in the analysis. The northern limit of oaks in Europe seems to be related to climatic factors that vary with latitude. On a coarse scale, *Q. petrea* follows the −4°C isotherm for the coldest month, whereas *Q. robur*, which reaches a little further north in Scandinavia, seems to be more limited by respiration sums (i.e., temperatures during the growing season, weighted by their effect on respiration; Dahl, 1998). Elevation and local topography may therefore also be important predictors of oak presence, as temperature generally declines with elevation and south‐facing slopes may have a considerably better local climate than north‐facing slopes at the same latitude and elevation (Stokland & Halvorsen, 2011). Both oak species seem to tolerate relatively dry habitats (Elven, 2005; Jones, 1959), and their recruitment is limited by light (Annighöfer et al., 2015). These factors may differ strongly between forest types and forestry regimes. Thus in forests, oaks are related to a combination of processes and variables associated with climate, topography, and land cover.

The distribution of veteran oaks, that is large and/or hollow oaks, may differ from the distribution of oak in general, because the development of large trees and tree hollows requires a long time without major disturbances. Most of the productive oak forests in Southern Norway have been heavily exploited for timber production. Large‐scale logging of oak forest in Norway started already in the 1,500s, peaked around 1,650—then the oak forests closest to the coast were heavily exploited and logging moved inland—and continued on a reduced scale until approximately 1,900 (Moore, 2010; Vevstad, 1998). The past 100 years or so, little logging of large oaks has taken place, but there has not been enough time for old‐growth oak forests to develop. In modern forestry, many oak forests have been logged and replaced by faster‐growing Norway spruce. It is unclear; however, to what extent veteran forest oaks are now concentrated in remote, inaccessible, and/or low‐productive forest areas.

Veteran oaks are also found in open landscapes. Oaks recruit naturally from trees within the open landscape or nearby forest. However, oaks are also of significant cultural importance and have been planted in courtyards, gardens and parks, and along roads and field margins. As in forests, oaks may have suffered from intensification of production in the agricultural landscape, where edge habitat and other marginal areas have been sacrificed in the creation of larger, more homogeneous production areas. In gardens and urban areas, oaks and other park trees are subject to various pressures from changing gardening trends and preferences related to safety.

### 2.2 Data

**Figure 2:**
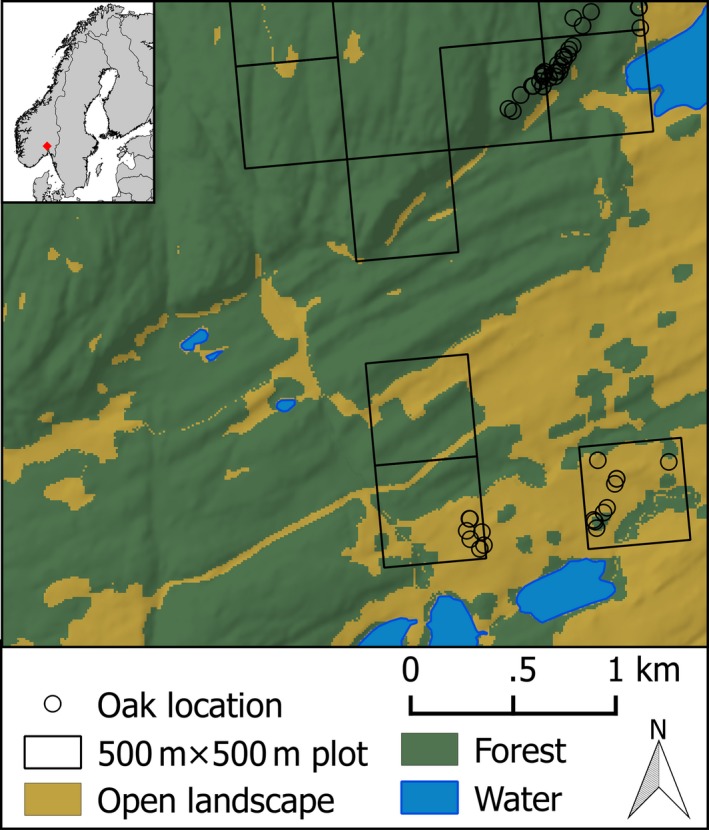
Selection of 500 × 500 m sample plots in a topographically diverse area with a mix of forest and open landscape types in Asker, Norway

We used data on veteran oak presences and absences from the pilot study behind the recently established national monitoring of veteran oaks (Sverdrup‐Thygeson et al., 2013). The data were collected as a stratified random sample within seven regions of approximately 200 km^2^ subjectively selected to represent the variation in the oak region in south‐eastern Norway. Each region was divided into 3 × 3 km blocks. We first randomly selected three blocks, and then 20 plots of 500 × 500 m within each block (Figure 2). Based on existing knowledge on occurrence of hollow oaks (primarily the database of the Norwegian Environment Agency; http://kart.naturbase.no/) we sorted the plots into “oak present”‐plots and “oak presence unclear”‐plots. All “oak present”‐plots and 25% of the “oak presence unclear”‐plots were visited in the field, and the position of all large and hollow oaks in the plot was determined with a hand‐held GPS. To prepare for analysis, each plot was gridded to obtain information on the presence and absence of veteran oaks in 10 × 10 m cells matching the geographical resolution of the predictors.

Potential predictors were collected from digital maps in a geographical information system (GRASS Development Team, 2015). Because the goal was prediction of oak presence across the landscape, we could only use variables for which (more or less) full‐cover maps were available. The variables considered were of two main kinds: (i) Geographical variables, reflecting large‐scale geographical gradients (e.g., latitude, longitude, elevation) and local topography (e.g., slope and aspect), and (ii) land cover variables, reflecting vegetation, landscape structure and human land use (e.g., forest, open landscape, distance to roads). When relevant, we considered variables at different spatial scales. Based on the literature and initial screening of about 80 potential predictor variables, we decided to test a set of 13 variables (Table 1) representing more or less independent aspects that could potentially affect the occurrence of veteran oaks (e.g., Dahl, 1998; Elven, 2005; Jones, 1959; McEwan et al., 2011; Moore, 2010; Stokland & Halvorsen, 2011; Vevstad, 1998), and for which data with more or less full geographical cover could be obtained (see Appendix S1 for further details on the variable screening process).

**Table 1:** Predictor variables and related patterns and processes

No.	Variable	Definition (units/scale)	Related patterns and processes
1	*X*	West–east coordinate (m)	Oceanic‐continental (moisture) gradient
2	*Y*	South–north coordinate (m)	Nemoral–boreal (temperature) gradient
3	*Z*	Elevation above sea level (m)	Correlated with air temperature, marine deposits, land use, etc.
4	*S*	Slope (degrees)	Correlated with insolation time, ground conditions and forestry activity
5	*A*	Aspect (northness)	Correlated with radiation sum
6	TWI	Terrain wetness index	Wetness indicator based on terrain and water flow from above
7	*K*	Fishers K (100 m radius)	Expresses terrain ruggedness, i.e. topographic variability
8	FA	Area of forest (patches >20 m across) within 1 km	Negatively related to intensive agriculture and urban areas
9	FD	Distance to forest edge (m)	Correlated with distance to open landscape
10	RD	Distance to road (m; water as a hard barrier)	Related to ease of access for logging, and for ornamental plantings (e.g. avenues)
11	WD	Distance to water (m)	Related to ease of access for past logging
12	*T*	Dominant tree class (T31: spruce, T32: pine, T33: deciduous)	Dominant tree species in forests
13	*P*	Productivity class (site index; P12_13: 12–13 m, etc.)	Represents productivity as reflected in height of the dominant tree species

For statistics on the variables in the different data sets, see Appendix S1: Table S1.

To test the prediction of different processes operating in different landscape types, we subdivided our study area into forest and open landscape types based on the predominant land cover. Forest landscapes were defined as all forest area with a patch size of >1 ha, and open landscapes were defined as all nonforest areas (excluding water and traffic area) plus forest patches with a size of 1 ha or less (see Appendix S1 for further details on the transition between forests and open landscapes).

### 2.3 Analysis

We developed prediction models for the full data set across all landscape types and for each landscape type separately by means of logistic regression (GLMs; McCullagh & Nelder, 1989). Visual inspection of the data indicated that some of the predictors could be nonlinearly related to oak presence. However, we found no support for strong nonlinearities in the initial model testing (GAM yielded essentially the same linear models as GLM), except for one predictor (slope), where the inclusion of a squared term (slope^2^) was necessary to account for a nonlinear response.

We developed a suite of models to elucidate key patterns and ensure robustness of the results with respect to our predictions. We analyzed a few single regression models with selected key predictors to test for human‐modified ecological responses (prediction 1) and different effects in forests and open landscapes (prediction 2). To evaluate the relative importance of these and other predictor variables we developed multiple regression models with several predictors. To evaluate model uncertainty and assess the need for multiple models (prediction 3) we also calculated AICc‐weighted average parameter estimates across multiple alternative models (Burnham & Anderson, 2002), and compared those to the best model (in terms of AICc). Because we wanted to focus on the effects of shifting human influence, we kept the geographical variables (number 1–7; Table 1) in all models and considered all possible combinations of the land cover variables (number 8–13). Model predictions were compared using receiving operator characteristics (area under the curve, AUC). Statistical modeling was carried out in R (R Core Team, 2014) and geographical prediction in GRASS GIS 7.0 (Grass Development Team, 2015).

## 3 RESULTS

We found that the distribution of veteran oaks was influenced by a mixture of ecological and anthropogenic factors, and that different processes dominated in forests and open landscapes.

**Figure 3:**
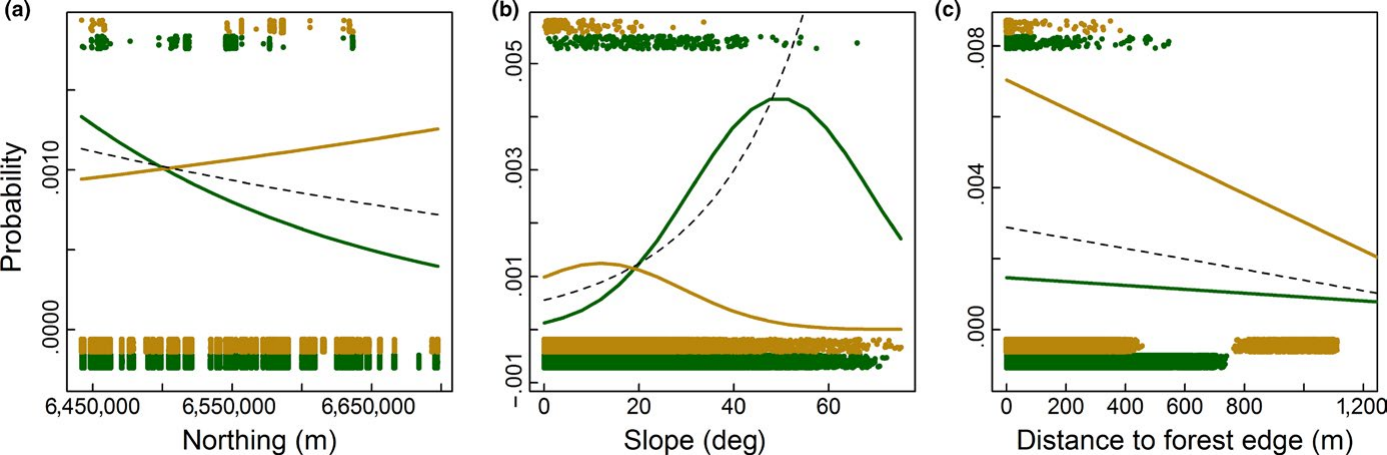
The probability of occurrence of veteran oaks as functions of (a) position along the latitudinal gradient, (b) local terrain slope, and (c) distance to forest edge. Data points on veteran oak occurrence in forest (green) and open landscapes (yellow) are plotted at the top (presences) and bottom (absences) of each panel (slightly spread out for clarity). Lines show predictions of fitted single logistic regression models for forests (green) and open landscapes (yellow), and both landscape types combined (dashed)

In accordance with the ecology and distribution of oaks in general, the probability of occurrence of veteran oaks declined toward the north in forests (Figure 3a, green line). However, in open landscapes, the pattern was opposite: the occurrence of veteran oaks increased toward the north (Figure 3a, brown line). Consequently, in the far north, a single‐predictor model based on data from both landscape types overestimated the probability of occurrence of veteran oaks in forests and under‐predicted the occurrence in open landscapes (Figure 3a, dashed line).

In addition to large‐scale geographical gradients, local topography was clearly important to the occurrence of veteran oaks. The probability of occurrence showed unimodal patterns with terrain slope in both forests and open landscapes, but shifted toward considerably steeper areas in forests than open landscapes (Figure 3b). The decline in occurrence toward very steep areas captured by both of the landscape‐specific models was missed when the data were combined: the single‐predictor model for the combined data set seriously over‐predicted occurrence in steep areas (Figure 3b, dashed line).

Landscape structure also affected probability of occurrence. In both forests and open landscapes, the occurrence of veteran oaks declined at increasing distances from the forest edge (Figure 3c). However, in the open landscape, the probability of occurrence was much higher near the forest edge, and the decline much steeper with increasing distance to the forest edge. Consequently, the total model strongly under‐predicted occurrence near the forest edge in the open landscape and over‐predicted near the edge inside the forest (Figure 3c, dashed line).

When we broadened the perspective to look at regression models with multiple predictors, we found that several variables were important. Although no single combination of variables was clearly better than the others for any of the data sets (the best models receive relatively weak support with AIC weights <0.5, and the confidence sets of models consist of 4–18 models; Table 2), there was a good correspondence between the best models (Table 3) and coefficient estimates based on AIC‐weighted model averaging across the confidence set of models (Table 4).

**Table 2:** Logistic regression models for the presence of veteran oak for each landscape type

Landscape type	Models in conf. set	Model rank	Model *X* + *Y* + *Z* + *A* + *S* + *S* ^2^ + TWI + *K*+	*k*	AICc	ΔAICc	AICc weight
Forest	18	1	*T* + *P* + FA + FD	15	3942.62	0.00	0.22
		2	*T* + *P* + FA	14	3943.10	0.48	0.17
		3	*T* + *P* + FA + FD + WD	16	3944.49	1.87	0.08
		4	*T* + *P* + FA + FD + RD	16	3944.62	2.00	0.08
Open	4	1	FA + FD	11	1996.39	0.00	0.48
		2	FA + FD + RD	12	1997.77	1.38	0.24
		3	FA + FD + WD	12	1998.33	1.94	0.18
		4	FA + FD + WD + RD	13	1999.74	3.35	0.09
All data	4	1	FA + FD + WD + RD	13	6276.09	0.00	0.46
		2	FA + FD + RD	12	6276.67	0.57	0.35
		3	FA + FD + WD	12	6279.12	3.03	0.10
		4	FA + FD	11	6279.30	3.20	0.09

The table shows the four best models (based on AICc) for each landscape type, the number of parameters (*k*, including geographical parameters), and AICc statistics. All models include the eight geographical variables below the table header “Model” in addition to the variables listed (see Table 1 for variable definitions).

**Table 3:** Model coefficients of the best logistic regression models for each data set, based on AICc

	Forest	Open landscape	All data
Intercept	63.800	−28.857	46.805
*X*	0.927***	−0.163	0.684***
*Y*	−0.763***	0.305	−0.551***
*Z*	−0.400***	−1.419***	−0.476***
*S*	1.286***	−0.413*	0.684***
*S* ^2^	−0.652***	0.185˄	−0.170*
*A*	−0.645***	−0.059	−0.443***
TWI	−0.259**	−0.693***	−0.465***
*K*	−0.074	0.022	−0.033
FA	−0.163*	−0.920***	−0.475***
FD	−0.103	−0.801***	−0.412***
RD	–	0.000	0.127*
WD	–	0.000	−0.077˄
T32	1.928***	–	–
T33	1.343***	–	–
P12_13	−0.360*	–	–
P14_15	−0.591**	–	–

Coefficients are standardized by *SD* of the predictors (Table 1, Appendix S1: Table S1).

*p*‐values for coefficient estimates (*z*‐tests): ***<.001, **<.01, *<.05, ˄<.1. (See Appendix S1: Table S2, for extended results.)

**Table 4:** Model coefficients averaged across the 95% confidence set of logistic regression models for each data set and standardized by the *SD* of the predictor variables (Table 1, Appendix S1: Table S1)

	Forest	Open landscape	All data
Intercept	59.978	−30.180	48.810
*X*	0.902^a^	−0.175	0.687^a^
*Y*	−0.724^a^	0.318	−0.573^a^
*Z*	−0.435^a^	−1.430^a^	−0.458^a^
*S*	1.263^a^	−0.410^a^	0.683^a^
*S* ^2^	−0.636^a^	0.183	−0.168
*A*	−0.648^a^	−0.059	−0.443^a^
TWI	−0.259^a^	−0.697^a^	−0.460^a^
*K*	−0.072	0.025	−0.036
FA	−0.174^a^	−0.919^a^	−0.483^a^
FD	−0.109	−0.801^a^	−0.404^a^
RD	−0.013	0.069	0.125^a^
WD	−0.053	0.020	−0.076
T32	1.949^a^	–	–
T33	1.348^a^	–	–
P12_13	−0.357^a^	–	–
P14_15	−0.587^a^	–	–

a Coefficients with 95% confidence intervals not including zero (see Appendix S1: Table S3, for extended results).

As expected, the probability of occurrence of veteran oaks declined with elevation in both forests and open landscapes. In addition to the elevational and latitudinal gradients there was a longitudinal gradient in occurrence in forests, with higher probability toward continental (dry) areas, but this effect was much weaker and even opposite in some models for open landscapes (Tables 3 & 4).

Several local topographic aspects were also important. The multiple regression models suggested a unimodal pattern for slope in forest (as in the single regression model; Figure 3b), as well as for all landscape types combined. The probability of occurrence was higher in dry parts of the landscape (negative effect of Terrain wetness index [TWI]) in both forests and open landscapes. Aspect (northness) had a negative effect, though only consistent across models in forest. Local topographic variability (Fischer's K) seemed to play a minor role in both systems.

Different aspects of land cover influenced the occurrence of veteran oaks in forests and open landscapes. In forests, the probability of occurrence was higher in deciduous and pine‐dominated stands than in spruce stands, and lower in highly productive areas. Other land cover variables played a minor role in forests. In open landscapes, on the other hand, the occurrence of veteran oaks was negatively related to the area of forest nearby and to distance to forest edge (as in the single regression model; Figure 3c).

**Figure 4:**
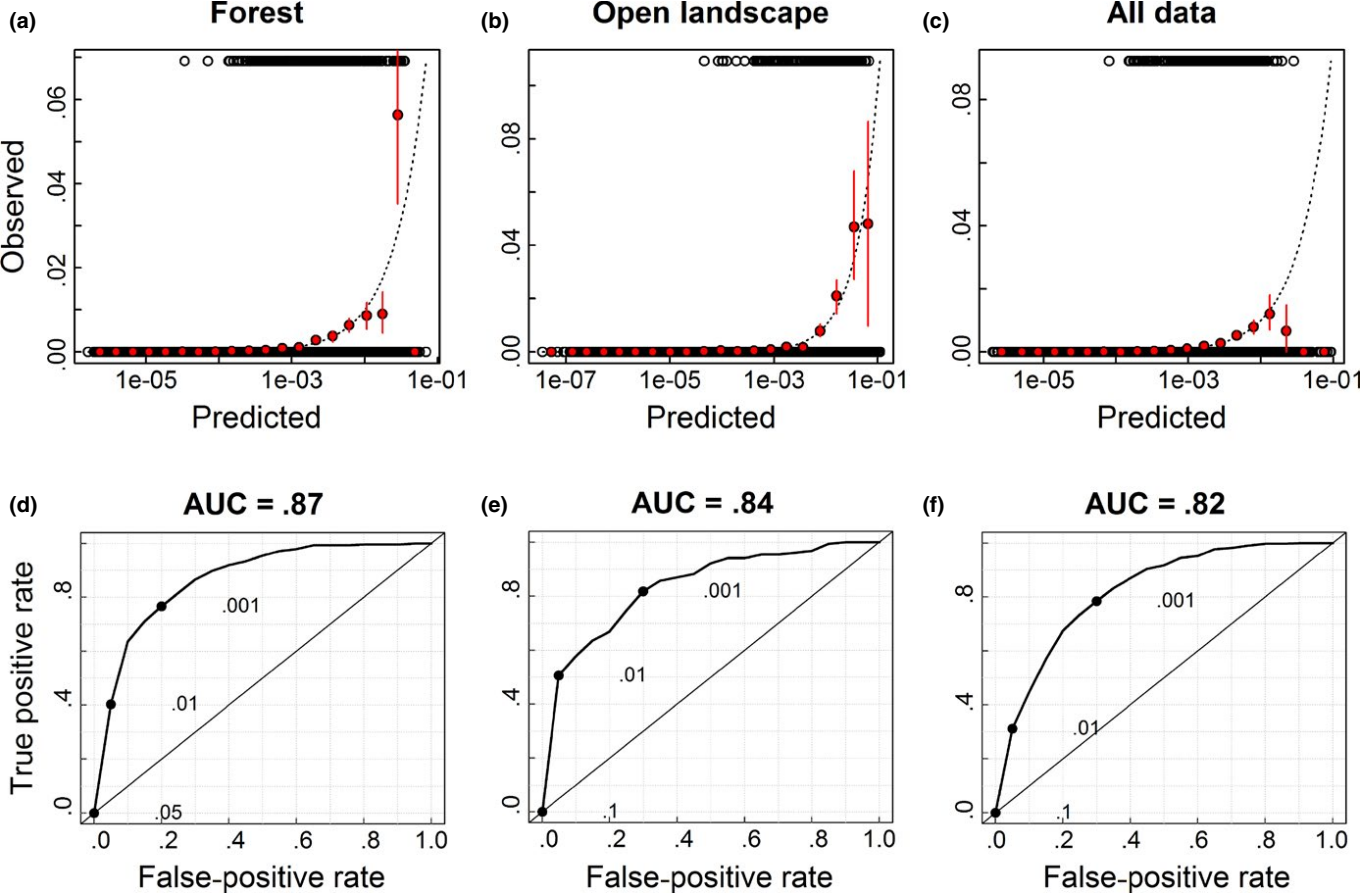
Validation plots for the best prediction models for all data, forests and open landscapes, showing observed versus predicted probability of occurrence (a,b,c) and the corresponding receiver‐operator curves (ROCs; d,e,f). The upper panels show presences (top) and absences (bottom) of veteran oak and means and confidence intervals of observations in red. The ROC‐plots show false predictions plotted against correct predictions for a range of cutoff values of predicted probabilities of occurrence, giving the area under the curve (AUC) as an indicator of predictive capacity

**Figure 5:**
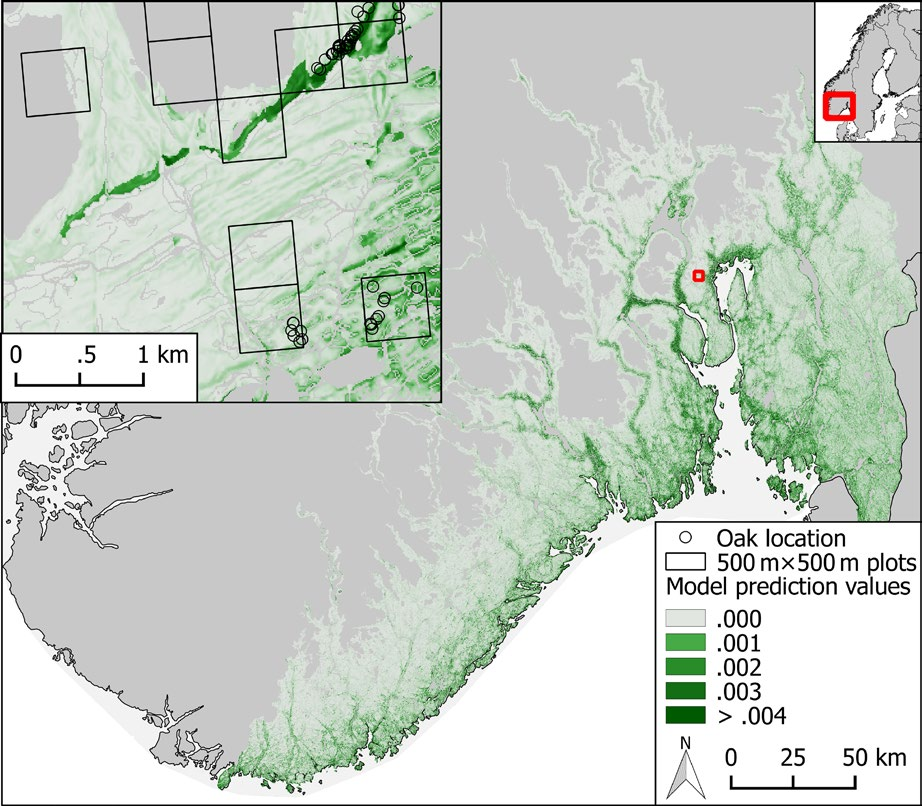
Maps of predicted probability of occurrence of veteran oaks (forest and open landscape models combined) showing the entire study area in *SE* Norway and a selected in Asker (inset map; corresponding to Figure 2)

Overall, predictions were better for landscape types separately than for all data combined and slightly better in forests than in open landscapes (Figure 4). Although the estimated probabilities of veteran oaks were generally low, the models clearly distinguished parts of the landscape with low and high (relatively speaking) probabilities of occurrence (Figures 4 and 5). However, while predictions were unbiased for models based on the landscape types separately the predictions based on all data overestimated the highest probabilities of occurrence (Figure 4). Geographical predictions were in agreement with the distribution of oak on a large scale, but anthropogenic factors were important for local variation in the probability of veteran oak occurrence (Figure 5).

## 4 DISCUSSION

A veteran oak is not just any forest tree. Veteran oaks are essential carriers of biodiversity, and of rich cultural traditions, like many other biodiversity hotspots (Habel et al., 2013; Lindenmayer & Laurance, 2016; Myers et al., 2000; Timonen et al., 2011). Moreover, like an increasing number of small and local hotspots, they are far apart, difficult to locate, and strongly influenced by anthropogenic processes. Developing models to predict their occurrence is, therefore, critically important and timely, but challenging. In both landscape types, the probability of encountering veteran oaks is low—even in the best spots. Our forest model predicts probabilities of about 0.05 for the best 10% of 10 × 10 m cells. This is still high compared to the expected probability for an average 10 × 10 m cell. On the basis of the same data, we have previously estimated that there are some 60,000 veteran oaks in Norway (Sverdrup‐Thygeson et al., 2011), distributed over 41,000 km^2^. This gives roughly 1.5 veteran oaks per km^2^, or an average probability of encountering a veteran oak in a 10 × 10 m cell of about 0.00015. Thus, despite low maximal probability, our models suggest that the odds are >300 times that of an average cell in the best spots. In other words, the models clearly distinguish hot and cold parts of the landscape.

Our results suggest that prediction of veteran oaks is possible, but also illustrate the importance of considering landscape structure as well as accounting for the different critical ecological and anthropogenic processes operating in natural and human‐dominated landscapes. Specifically, our predictions and results highlight three major challenges for ecological and geographical prediction in the Anthropocene.

First, biodiversity hotspots may respond differently to ecological factors (biotic and abiotic) in different landscapes depending on the kind and degree of human influence (prediction 1). Examples include differences in magnitude and steepness of linear responses, direction of linear responses, and position of realized optima along ecological gradients. This is clearly seen in the different responses of veteran oaks to forest edge, latitude, and slope, respectively (Figure 3). Such shifts may be induced by varying human influences along the gradients in different landscape types, generating a greater difference between the realized and the fundamental niche in one landscape type than in another. For instance, intensive agriculture and urban development may have reduced the occurrence of veteran oaks in the south, while oaks may have been favored as exotic elements in the less intensively driven cultural landscape toward the north. In forests, these human factors play a lesser role, and the occurrence of veteran oaks declines toward the north, in concordance with the fundamental oak niche (Figure 3a). However, in forests, harvesting is difficult in steep areas, where veteran oaks consequently have a strongly elevated probability of occurrence compared to similar slopes in the open landscape (Figure 3b). This aligns well with the general overrepresentation of key habitats for biodiversity in steep terrain in forests (Sætersdal, Gjerde, Heegard, Schei, & Nilsen, 2016) and with our previous work suggesting that the diversity and species composition in oak hot spot habitats differ in open landscapes and forests, and respond to different factors in these two systems (Sverdrup‐Thygeson, Skarpaas, & Ødegaard, 2010). When responses differ strongly between landscape types, as in the veteran oak examples, simply adding landscape type as another covariate in a (generalized) linear regression model is not enough to resolve the problem. Interaction terms between landscape type and other predictors could help account for different responses to common predictors in the different landscape types. However, this is only possible for predictors that are relevant in all landscape types where the biodiversity hot spot occurs.

This brings us to the second general challenge for spatial prediction modeling: different predictors may be relevant only in specific areas (prediction 2). Humans frequently modify ecosystems to the extent that original natural processes are no longer the most relevant and important processes structuring ecosystems (Ellis et al., 2010). For instance, for veteran oaks (and other old trees; see Lindenmayer & Laurance, 2016; Sætersdal et al., 2016), dominant tree species, and forest productivity are important variables in forests, but not relevant in the open landscape where the forest has been cleared and kept away, often for centuries. This implies that the sets of relevant and important predictor variables are different in the two landscape types. One solution to this problem is to split the ecological predictions by landscape type, as we did here (see also Meineri, Skarpaas, & Vandvik, 2012): landscape‐specific models can be developed for different landscape types, and predictions merged geographically (i.e., on a map). This approach requires that landscape types are clearly defined and that information on the spatial distribution of landscape types is readily available.

Finally, the third challenge highlighted by our study is the multitude and complexity of processes and factors affecting biodiversity hotspots in human‐influenced landscapes (prediction 3). Ecological processes are complex, anthropogenic processes even more so. This challenge goes far beyond the time‐ and space‐dependent relevance and importance of single‐predictor variables discussed above. It is clear from our study of veteran oaks that no predictor can be singled out as the one and only or clearly most important one. Multiple predictors are needed, representing both ecological and anthropogenic processes. This is in accordance with the “multiple ecosystem drivers hypothesis” of oak forest dynamics (McEwan et al., 2011), although the oak species and drivers are different in our European system than in North America (e.g., no sudden oak death in Europe) and our study concerns veteran oaks in particular. We know several of the factors affecting the life and death of oaks, and the conditions for generating tree hollows, yet we are far from a solid understanding of all major processes and their interactions, and lack data on relevant variables. For veteran oaks, as for most biological systems, we have to make do with relatively simple data and statistical modeling tools. In this situation, there is potentially a high degree of model uncertainty. To address model uncertainty, we presented and combined several models based on AIC weights (Burnham & Anderson, 2002; for alternative methods for averaging model predictions, see e.g., Romero, Olivero, Brito, & Real, 2015). Our analysis shows that although a number of alternative models can predict the observed occurrences of veteran oaks almost equally well, the predictors and parameter estimates of the best models are also given strong support when combining multiple models. Thus, while testing several alternative models provides a broader basis for inference (but see e.g., Cade, 2015 for pitfalls), multiple models are not necessary for parameter estimation and prediction in this case.

Underlying all of the three modeling challenges above is the quantification of anthropogenic processes. Development of relevant and measurable predictors is critically important in an increasingly human‐dominated world. Unfortunately, there are large knowledge gaps related to land use (Erb et al., 2017). Historical land use and landscape development are often poorly documented in maps, especially in digital maps, both in open landscapes and in forests. In this study we therefore, screened a large number of potential proxies derived from current land cover and structural landscape variables related to past and present human activities of relevance to veteran oaks. We arrived at a limited set of variables selected to represent key processes (e.g., forest area, distance to road and distance to water; Table 1). Veteran oaks respond negatively to forest area in the surroundings, both in forests and in open landscapes (Tables 3 & 4). This suggests that veteran oaks in open landscapes may be found in urban parks and productive agricultural areas, whereas veteran oaks in forests may be associated with marginal areas for both forestry and agriculture. Many of the large oaks growing in forest today may have been growing in open landscapes in a not too distant past. Even marginal areas in Norway were heavily exploited for fuelwood, grazing etc. about a hundred years ago (Framstad & Lid, 1998), but many oaks may have been spared as ornamental trees, especially toward higher elevations where oaks are less common (see further discussions of transitional landscapes in Appendix S1). Thus, the forest area variable seems to capture anthropogenic landscape structures and processes of importance to veteran oaks. Distance to road and water, on the other hand, did not have significant predictive power (Tables 3 & 4). This result was unexpected, as timber is largely transported by road in present‐day forestry, whereas log driving on rivers and lakes was the main means of moving timber from the forest to the sawmills in the past (Sandmo, 1951). Steepness (and productivity) of the terrain seem to better represent effects of logging activities on oak than distance to roads and water. However, direct data on past logging activities would clearly have been much more informative than proxies based on current landscape structures. This underscores the need for keeping track of major human land use activities for future studies of their effects, especially for systems involving slow ecological processes.

Despite the challenges discussed above, our spatial models for veteran oaks provide clear results of relevance of biodiversity management and conservation as well as further research. It is evident from our results that veteran oaks are influenced by more than the natural factors shaping the fundamental oak niche. Our models suggest that elevation, terrain wetness, and landscape structure (forest area) are important predictors of veteran oak presence, in accordance with the fundamental oak niche (Dahl, 1998; Jones, 1959; Stokland & Halvorsen, 2011), but with differing responses and additional variables playing major roles in forested and open landscape types, as discussed above. We now have a workable set of prediction models that can help us design mapping and monitoring efforts, improve estimates of veteran oak abundance, guide conservation management (Lindenmayer et al., 2014), and support research addressing issues such as cost‐effective probability‐based sampling (Yoccoz, Nichols, & Boulinier, 2001), effects of landscape structure and connectivity (Evju, Blumentrath, Skarpaas, Stabbetorp, & Sverdrup‐Thygeson, 2015; Evju & Sverdrup‐Thygeson, 2016; Sverdrup‐Thygeson, Skarpaas, Blumentrath, Birkemoe & Evju, [Link]in press), predictions of species richness (Skarpaas et al., 2011), and spatial community dynamics (Engen, Sæther, Sverdrup‐Thygeson, Grøtan, & Ødegaard, 2008).

To summarize and conclude, we find that veteran oaks are predictable despite the complexity of processes in human‐influenced landscapes and that considering how different human‐related processes operate in different landscapes helps both understanding veteran oak responses to environmental variables and prediction of distribution patterns. We expect predictions to be further improved with the extensive monitoring data set under establishment, especially after repeated visits. This will document recruitment and mortality patterns in veteran oaks and can be used to develop increasingly refined process‐oriented models. Regardless of the modeling approach, finding ways to account for human influence on ecological systems and address the challenges illustrated by the veteran oak case are likely to become increasingly important in the Anthropocene.

## CONFLICT OF INTERESTS

None declared.
