# Supplementary material for: Prediction of biodiversity hotspots in the Anthropocene: The case of veteran oaks
Source: Ecol Evol. 2017 Aug 31;7(19):7987–97. doi: 10.1002/ece3.3305 (PMC5632640; doi:10.1002/ece3.3305)
Supplement: Supplementary file 1 [file ECE3-7-7987-s001.docx]

Supplementary material for

Prediction of biodiversity hotspots in the Anthropocene:

The case of ancient oaks

Olav Skarpaas, Stefan Blumentrath, Marianne Evju and Anne Sverdrup-Thygeson

# Appendix S1. Extended Methods and Results

In this Appendix we report additional details on landscape types, predictor variables and results to broaden the basis of our study.

## Landscape types and predictors

In the main article we focus on two landscape types: (1) forest landscapes, defined as all forest area with a patch size of > 1 ha, and (2) open landscapes, defined as all non-forest areas (excluding water and roads) plus forest patches with a size of 1ha or less. Here, we also consider a third landscape type: (3) transition landscapes, defined as tree-covered areas of 1-2 ha or with a width of not more than ca. 100m. The transition landscapes were included in forest in the main article. Here we report results for strict forest (i.e. without transition), forest (with transition), and transition separately, in addition to open landscape.

As described in the main article, potential predictors were collected from digital maps in a geographical information system (GRASS Development Team 2015). We screened about 80 predictor variables representing large-scale geographical gradients and topography and land cover at several different scales. We explored the correlations between the variables (including the response variable) and assessed independence and predictions to reduce the number of predictors. For instance, we considered several variables representing solar radiation, but left them out of the final analysis: insolation time (spring/summer) and total radiation sum (spring/summer) are highly correlated with slope and aspect respectively. Ground condition (soil, rocks) was also left out because it was correlated with topographic variables (mainly slope). Terrain position index (TPI) was considered, but left out as it correlated with Fischer’s K at several scales. Properties related to the open landscape (distance to open landscape, area of open landscape) that were negatively related to forest properties (distance to forest edge, forest area) were left out. Finally, in forests, dominant tree species was selected because it was a better predictor than satellite-based volume estimates of the tree species. After the screening process we arrived at a set of 13 more or less independent predictor variables (see Table 1 in main article).

We developed prediction models (as described in the main article) for all combinations of data sets and landscape types. Because the landscape types and data sets are different samples of the entire landscape, the means and standard deviations of predictor variables differ somewhat among the data (sub)sets (Table A1). Standard deviations were used to standardize variables before analysis.

## Extended results for different landscape types

For large-scale geographical variables (X, Y, Z) the coefficients for the transitional landscape are larger (in absolute value) than for forests and open landscapes (Tables A2 & A3), suggesting a greater decline in ancient oak occurrence towards the north, but a higher increase towards the east and towards higher elevations in transitional landscapes compared to strict forests and open landscapes. This supports the idea that ancient oaks are favoured in the marginal areas production landscape, up from the coast. This is also supported by the strong negative effect of forest area and the positive effect of tree species other than spruce (T32 pine and T33 deciduous). For most topographical variables (slope, aspect, TWI) the results for the transitional landscape are intermediate between forests and open landscape.

## References

GRASS Development Team. 2015. Geographic Resources Analysis Support System (GRASS) Software, Version 7.0. - Open Source Geospatial Foundation.

Table S1. Mean and SD of predictor variables (see Table 1 in main article for definitions) for the two data sets in each landscape type (see Appendix text for definitions).

|  | Strict forest | | |  | Forest (incl. Transition) | | |  | Transition | | |  | Open landscape | | |  | All data | | |
| --- | --- | --- | --- | --- | --- | --- | --- | --- | --- | --- | --- | --- | --- | --- | --- | --- | --- | --- | --- |
|  | Mean |  | SD |  | Mean |  | SD |  | Mean |  | SD |  | Mean |  | SD |  | Mean |  | SD |
| X | 528089 |  | 56769 |  | 528582 |  | 56935 |  | 556365 |  | 59334 |  | 559250 |  | 58453 |  | 536266 |  | 58840 |
| Y | 6552572 |  | 63291 |  | 6553007 |  | 63436 |  | 6577510 |  | 66745 |  | 6575350 |  | 64220 |  | 6558604 |  | 64366 |
| Z | 202 |  | 120 |  | 200 |  | 120 |  | 99 |  | 77 |  | 98 |  | 88 |  | 174 |  | 122 |
| S | 12,90 |  | 9,82 |  | 12,85 |  | 9,79 |  | 9,56 |  | 7,25 |  | 5,40 |  | 5,63 |  | 10,98 |  | 9,49 |
| S^2^ | 262,89 |  | 395,42 |  | 260,82 |  | 393,26 |  | 143,98 |  | 211,34 |  | 60,89 |  | 168,31 |  | 210,73 |  | 361,26 |
| A | -0,003 |  | 0,685 |  | -0,002 |  | 0,685 |  | 0,072 |  | 0,719 |  | -0,046 |  | 0,713 |  | -0,013 |  | 0,693 |
| TWI | 6,026 |  | 1,643 |  | 6,031 |  | 1,646 |  | 6,311 |  | 1,806 |  | 7,448 |  | 1,737 |  | 6,386 |  | 1,779 |
| K | 0,309 |  | 0,223 |  | 0,310 |  | 0,224 |  | 0,347 |  | 0,234 |  | 0,278 |  | 0,243 |  | 0,302 |  | 0,229 |
| FA | 25690 |  | 4856 |  | 25511 |  | 5077 |  | 15436 |  | 6797 |  | 14240 |  | 7829 |  | 22687 |  | 7650 |
| FD | 4,250 |  | 1,350 |  | 4,209 |  | 1,384 |  | 1,912 |  | 1,312 |  | 3,454 |  | 1,280 |  | 4,020 |  | 1,397 |
| RD | 5,054 |  | 1,354 |  | 5,030 |  | 1,367 |  | 3,696 |  | 1,433 |  | 3,408 |  | 1,687 |  | 4,624 |  | 1,615 |
| WD | 8,100 |  | 0,834 |  | 8,107 |  | 0,839 |  | 8,524 |  | 0,982 |  | 8,064 |  | 1,087 |  | 8,096 |  | 0,907 |

Table S2. Model coefficients of the best models for each data set, based on AICc. Coefficients were standardized by the SD of the predictor variables (Table S1). P-values for coefficient estimates (z-tests): *** < 0.001, ** < 0.01, * < 0.05, . < 0.1.

|  | Strict forest |  |  | Forest (incl. Transition) |  |  | Transition |  |  | Open landscape |  |  | All data |  |
| --- | --- | --- | --- | --- | --- | --- | --- | --- | --- | --- | --- | --- | --- | --- |
| Intercept | 56,426 |  | | 63,800 |  | | 357,106 |  | | -28,857 |  |  | 46,805 |  |
| X | 0,854 | *** | | 0,927 | *** | | 3,007 | ** | | -0,163 |  |  | 0,684 | *** |
| Y | -0,690 | *** | | -0,763 | *** | | -4,048 | ** | | 0,305 |  |  | -0,551 | *** |
| Z | -0,516 | *** | | -0,400 | *** | | 2,921 | *** | | -1,419 | *** | | -0,476 | *** |
| S | 1,355 | *** | | 1,286 | *** | | 0,701 |  |  | -0,413 | * |  | 0,684 | *** |
| S^2^ | -0,699 | *** | | -0,652 | *** | | -0,427 |  |  | 0,185 | . |  | -0,170 | * |
| A | -0,700 | *** | | -0,645 | *** | | 0,201 |  |  | -0,059 |  |  | -0,443 | *** |
| TWI | -0,299 | ** | | -0,259 | ** | | 0,294 |  |  | -0,693 | *** | | -0,465 | *** |
| K | -0,102 |  |  | -0,074 |  |  | 0,359 |  |  | 0,022 |  |  | -0,033 |  |
| FA | - |  |  | -0,163 | * |  | -1,298 | ** | | -0,920 | *** | | -0,475 | *** |
| WD | - |  |  | - |  |  | 0,000 |  |  | 0,000 |  |  | -0,077 | . |
| RD | - |  |  | - |  |  | -0,392 | . |  | 0,000 |  |  | 0,127 | * |
| FD | -0,116 | . |  | -0,103 |  |  | 0,000 |  |  | -0,801 | *** | | -0,412 | *** |
| T32 | 1,872 | *** | | 1,928 | *** | | 6,086 | * |  | - |  |  | - |  |
| T33 | 1,213 | *** | | 1,343 | *** | | 6,231 | * |  | - |  |  | - |  |
| P12_13 | -0,295 | . |  | -0,360 | * |  | - |  |  | - |  |  | - |  |
| P14_15 | -0,566 | ** | | -0,591 | ** | | - |  |  | - |  |  | - |  |

Table S3. Model coefficients averaged across the 95%-confidence set of models for each data set and standardized by the SD of the predictor variables (Table S1). * Coefficients with 95%-confidence intervals not including zero.

|  | Strict forest | |  | Forest (incl. transition) | |  | Transition | |  | Open landscape | |  | All data |  |
| --- | --- | --- | --- | --- | --- | --- | --- | --- | --- | --- | --- | --- | --- | --- |
| Intercept | 55,127 |  |  | 59,978 |  |  | 368,156 |  |  | -30,180 |  |  | 48,810 |  |
| X | 0,848 | * |  | 0,902 | * |  | 3,117 | * |  | -0,175 |  |  | 0,687 | * |
| Y | -0,676 | * |  | -0,724 | * |  | -4,180 | * |  | 0,318 |  |  | -0,573 | * |
| Z | -0,528 | * |  | -0,435 | * |  | 2,977 | * |  | -1,430 | * |  | -0,458 | * |
| S | 1,350 | * |  | 1,263 | * |  | 0,621 |  |  | -0,410 | * |  | 0,683 | * |
| S^2^ | -0,691 | * |  | -0,636 | * |  | -0,365 |  |  | 0,183 |  |  | -0,168 |  |
| A | -0,704 | * |  | -0,648 | * |  | 0,188 |  |  | -0,059 |  |  | -0,443 | * |
| TWI | -0,302 | * |  | -0,259 | * |  | 0,269 |  |  | -0,697 | * |  | -0,460 | * |
| K | -0,094 |  |  | -0,072 |  |  | 0,312 |  |  | 0,025 |  |  | -0,036 |  |
| FA | -0,059 |  |  | -0,174 | * |  | -1,278 | * |  | -0,919 | * |  | -0,483 | * |
| WD | -0,052 |  |  | -0,053 |  |  | 0,030 |  |  | 0,020 |  |  | -0,076 |  |
| RD | -0,015 |  |  | -0,013 |  |  | -0,392 |  |  | 0,069 |  |  | 0,125 | * |
| FD | -0,111 |  |  | -0,109 |  |  | 0,036 |  |  | -0,801 | * |  | -0,404 | * |
| T32 | 1,887 | * |  | 1,949 | * |  | 6,654 | * |  | - |  |  | - |  |
| T33 | 1,214 | * |  | 1,348 | * |  | 6,764 | * |  | - |  |  | - |  |
| P12_13 | -0,291 |  |  | -0,357 | * |  | -0,747 |  |  | - |  |  | - |  |
| P14_15 | -0,562 | * |  | -0,587 | * |  | -1,030 |  |  | - |  |  | - |  |
